# Lipid biomarkers of GVHD in allogeneic stem hematopoietic cell transplantation patients

**DOI:** 10.3389/fimmu.2025.1624168

**Published:** 2025-09-02

**Authors:** Zhi Lin, Jie Shen, Yicheng Fu, Jiao Liu, Lu Yan, Xin Li, Minghua Yang

**Affiliations:** ^1^ Department of Pediatrics, The Third Xiangya Hospital, Central South University, Changsha, Hunan, China; ^2^ Hunan Clinical Research Center of Pediatric Cancer, Changsha, China; ^3^ DAMP Laboratory, Department of Critical Care Medicine, Third Affiliated Hospital of Guangzhou Medical University, Guangzhou, Guangdong, China; ^4^ Department of Hematology, The Third Xiangya Hospital, Central South University, Changsha, Hunan, China

**Keywords:** lipid, HDL - cholesterol, HSCT = hematopoietic stem cell transplant, GVHD (graft-versus-host disease), GRFS

## Abstract

**Objective:**

While dyslipidemia is established as a key modulator of innate and adaptive immune responses, its role in hematopoietic reconstitution remains unclear. This study aimed to characterize lipid profiles in patients undergoing allogeneic hematopoietic stem cell transplantation (HSCT) and evaluate the associations between dyslipidemia and clinical outcomes.

**Methods:**

A retrospective analysis was conducted in a cohort of 106 adult patients (≥18 years) who underwent allogeneic HSCT between January 2019 and December 2023 and had complete lipid records.

**Results:**

Profound dyslipidemia was observed post-transplantation, with over 60% of patients developing significantly decreased high-density lipoprotein cholesterol (HDL-C) and elevated triglycerides (TG), total cholesterol (TC), and low-density lipoprotein cholesterol (LDL-C) compared to baseline. HDL-C reached its nadir around day 14 and recovered slowly thereafter. Patients with grade III–IV acute graft-versus-host disease (GVHD) exhibited significantly lower HDL-C levels compared to those without GVHD. Lower HDL-C levels were correlated with delayed neutrophil engraftment and inferior GVHD-free/relapse-free survival (GRFS), though not with overall survival (OS). An HDL-C threshold of ≤0.84 mmol/L was identified as an independent predictor of GVHD.

**Conclusion:**

Early post-transplant HDL-C dynamics represent a promising biomarker for GVHD risk stratification. These findings support the incorporation of protocolized lipid monitoring into HSCT management to guide preemptive therapeutic interventions.

## Introduction

Allogeneic hematopoietic stem cell transplantation (allo-HSCT) is an effective therapy for patients with aggressive hematological malignancies and nonmalignant hematological disorders. However, graft-versus-host disease (GVHD) remains a critical threat to survival post-transplantation. It is well known that the success of HSCT is mostly the result of achieving a balance between graft-versus-tumor and graft-versus-host effects. Controlling the alloimmune response to decrease relapse without increasing GVHD is a critical goal for improving disease control without increasing mortality. Hence, it is of great importance to recognize some variations that might play a role in regulating alloimmunity in HSCT recipients.

Many studies have concentrated on the potential role of statins in GVHD prophylaxis, but these studies have rarely focused on lipid levels (1, 2). Dyslipidemia affects both innate and adaptive immune reactions by modulating the release of proinflammatory cytokines, which contribute to the activation and polarization of T cells (3). Diet-induced dyslipidemia can induce trained innate immunity in hematopoietic stem and progenitor cells (HSPCs) characterized by epigenetic and metabolic reprogramming (4). High-density lipoprotein (HDL) is considered to play anti-inflammatory and antioxidative roles by suppressing immune cell chemotaxis and activation (5). Recently, it was reported that the absence of circulating HDL aggravated hepatic acute GVHD and increased the mortality of allogeneic transplant recipient model mice (6), highlighting the potential impact of dyslipidemia on GVHD severity. Conversely, GVHD itself is a well-established contributor to dyslipidemia. As the liver and intestines are primary targets of GVHD and are also central organs for lipid metabolism, GVHD-induced damage to these tissues can profoundly disrupt lipid homeostasis. Prior studies have demonstrated an increased prevalence of dyslipidemia after HSCT, but data are lacking on the contribution of dyslipidemia to clinical outcomes. Given this potential bidirectional relationship (where dyslipidemia may exacerbate GVHD and GVHD may worsen dyslipidemia, creating a deleterious cycle), and the recognized immunomodulatory impact of lipoproteins, we conducted a retrospective study to determine the incidence and illustrate the time course of dyslipidemia following HSCT and to identify associations between serum lipid profiles and clinical outcomes.

## Methods

### Patients

This study included 106 adult patients who underwent allo-HSCT at the Third Xiangya Hospital of Central South University between January 2019 and December 2023 and had complete longitudinal lipid profiles. Patients with underlying diseases of dyslipidemia or incomplete lipid data were excluded based on screening of medical records. This study was approved by the Ethics Committee of the Third Xiangya Hospital (Ethic committee, Third Xiangya Hospital, Central South University, No.23067). All participants provided written informed consent in accordance with the Declaration of Helsinki.

### Transplantation

The conditioning regimens were defined according to the consensus from The Chinese Society of Hematology (CSH) (7). Most patients with hematological malignancies received a standard myeloablative regimen of modified busulfan and cyclophosphamide. Elderly patients (age ≥ 55 years old) and patients with a high risk of comorbidity underwent a reduced-intensity regimen (RIC) in which cyclophosphamide was substituted (or partially substituted) with fludarabine. Patients with refractory leukemia underwent an intensified conditioning regimen. Additionally, anti-thymocyte globulin (ATG) was added to the conditioning regimen for mismatched unrelated donor transplantations or transplantations for nonmalignant diseases. All patients received graft-versus-host disease (GVHD) prophylaxis consisting of cyclosporine A (CsA), methotrexate (MTX), and mycophenolate mofetil (MMF).

### Definitions

Diagnoses and severity grading of acute GVHD (aGVHD) and chronic GVHD (cGVHD) followed the criteria outlined in the *Chinese Expert Consensus on Acute GVHD* (8) and *Chinese Expert Consensus on Chronic GVHD* (9), respectively. aGVHD comprises two distinct subtypes: (i) Classic aGVHD—manifesting inflammatory injury to the skin, gastrointestinal tract, and/or liver within 100 days post-transplant (+100 d); and (ii) Late-onset aGVHD—exhibiting identical diagnostic features but emerging >100 days post-transplant. Chronic GVHD (cGVHD) encompasses the broad spectrum of disease occurring beyond +100 d, not exclusively defined by temporal boundaries but rather by clinicopathological manifestations.

Neutrophil recovery was defined as the first of three consecutive days with ANC ≥0.5×10^9^/L, while platelet recovery required seven consecutive transfusion-free days with platelet counts ≥20×10^9^/L (10).

### Lipid profile

Lipid levels during HSCT, including HDL-C, LDL-C, TC and TG levels, were collected from electronic medical charts. The lipid levels were classified according to the normal limits recommended by the National Cholesterol Education Program (NCEP) guidelines (11). Dyslipidemia was defined as levels above or below the normal range for one or more lipids. Lipid values were defined as follows: low HDL‐C, <1 mmol/L in men and <1.3 mmol/L in women; high TG, >2.25 mmol/L; high TC, > 5 mmol/L, and high LDL-C, > 3.2 mmol/L.

### Statistical analysis

SPSS 26.0 and GraphPad Prism 8.0 were used for statistical analysis and drawing. Continuous variables are expressed as the median (range). The Mann–Whitney U test was used to evaluate differences in general characteristics and laboratory results between groups. Categorical variables are expressed as counts and proportions and were evaluated using the chi-squared test or Fisher’s exact test. The overall survival (OS) and GVHD-free/relapse-free survival (GRFS) were estimated by the Kaplan–Meier method and compared by the log-rank test.

Data analysis was performed in R v4.4.3 using generalized linear mixed models (GLMMs) to evaluate dynamic associations between high-density lipoprotein (HDL) levels and graft-versus-host disease (GVHD) probability over post-transplant time. After preprocessing the longitudinal dataset, converting patient IDs to factors, and applying square-root transformation to time variables, four GLMM variants were compared: 1) Base model (HDL + Time + random intercept); 2) Interaction model (HDL×Time); 3) Nonlinear model (HDL²); 4) Time-stratified model (early/late phase). Model selection via likelihood ratio tests and AIC/BIC minimization identified the interaction model as optimal, enabling quantification of time-dependent HDL effects. Final visualizations included trajectory plots at 8 timepoints with 95% CIs and 3D interaction surfaces, with residual diagnostics confirming model adequacy.

Cumulative incidence functions (CIFs) for GVHD were estimated using Fine-Gray proportional subdistribution hazards models via the R package cmprsk with GVHD and non-GVHD mortality as competing events. All patients were followed from transplantation until GVHD onset, competing event occurrence, or last contact. Event status was coded as: 0=censored, 1= GVHD, 2= GVHD and non-GVHD mortality. Subdistribution hazard ratios (sHR) with 95% confidence intervals quantified HDL effects on GVHD risk in the presence of competing events.

Univariate and multivariate logistic regression models were used to evaluate the relationships between lipid levels and GVHD, and unadjusted and adjusted odds ratios (ORs) and 95% confidence intervals (Cis) were calculated. In the multivariate adjusted models, sex, age and diagnosis (including acute lymphoid leukemia, acute myeloid leukemia (AML), aplastic anemia (AA), myelodysplastic syndrome (MDS), and other disease) were included. A *P* value < 0.05 (two-tailed) was considered statistically significant.

## Results

### Patient and transplantation characteristics

A total of 106 patients were enrolled in our study. The median age of the patients in the retrospective cohort was 39 years (range, 18–58 years). Of the patients, 64 (60.38%) had AML, 23 (21.7%) had acute lymphoid leukemia (ALL), 11 (10.37%) had MDS, 2 (1.89%) had AA, and 6 (5.66%) had other diseases. The median number of MNCs infused for the stem cell transplantation was 9.47×10^8^/kg (range, 0.87-19.9×10^8^/kg), and the median number of CD34+ cells infused was 4.37×10^6^/kg (range, 0.62-16.8×10^6^/kg). The median times to neutrophil and platelet engraftment were 11 (range, 8-19) and 13 (range, 7-46) days, respectively. Most patients had a matched related donor (n=86, 81.13%). The demographics are summarized in **Table 1**.

**Table 1:** Clinical characteristics of the HSCT patients.

Variable	Total (n=106)	Group		*P*	Group		*P*
		None (n=61)	GVHD (n=45)		Alive (n=83)	Dead (n=23)	
Median age, years (range)	39 (18~58)	39 (18~58)	39 (18-58)	0.8156	39 (18~58)	39 (18~58)	0.806
Sex, No. (%)				0.976			0.200
Male	52 (49.06)	31 (50.82)	23 (51.11)		45 (54.22)	9 (39.13)	
Female	54 (50.94)	30 (49.18)	22 (48.89)		38 (45.78)	14 (60.87)	
Diagnosis, No. (%)				0.554			0.019
ALL	23 (21.70)	13 (21.31)	10 (22.22)		16 (19.28)	7 (30.43)	
AML	64 (60.38)	40 (65.57)	24 (53.32)		54 (65.06)	10 (43.48)	
AA	2 (1.89)	1 (1.64)	1 (2.22)		0 (0.00)	2 (8.70)	
MDS	11 (10.37)	4 (6.56)	7 (15.56)		7 (8.43)	4 (17.39)	
Others	6 (5.66)	3 (4.92)	3 (6.67)		6 (7.23)	0 (0.00)	
Donor–recipient sex match, n (%)							
Female to male	13 (12.26)	8 (13.11)	5 (11.11)	0.756	11 (13.25)	2 (8.70)	0.818
Cells infused, n (range)							
MNCs, median, × 10^8^/kg	9.47 (0.87-19.90)	7.60 (1.05~19.90)	9.76 (0.87~18.00)	0.164	9.40 (1.05~19.9)	10.14 (0.87~18.00)	0.475
CD34^+^ cells, median, × 10^6^/kg	4.37 (0.62~16.80)	4.33 (0.62~7.98)	4.46 (1.00~16.80)	0.139	4.40 (0.62~16.80)	5.01 (1.98~12.70)	0.108

ALL, acute lymphoid leukemia; AML, acute myeloid leukemia; AA, aplastic anemia; MDS, myelodysplastic syndrome; MNCs, mononuclear cells; GVHD, graft-versus-host disease.

### Serum lipid profiles during HSCT

**Figure 1:**
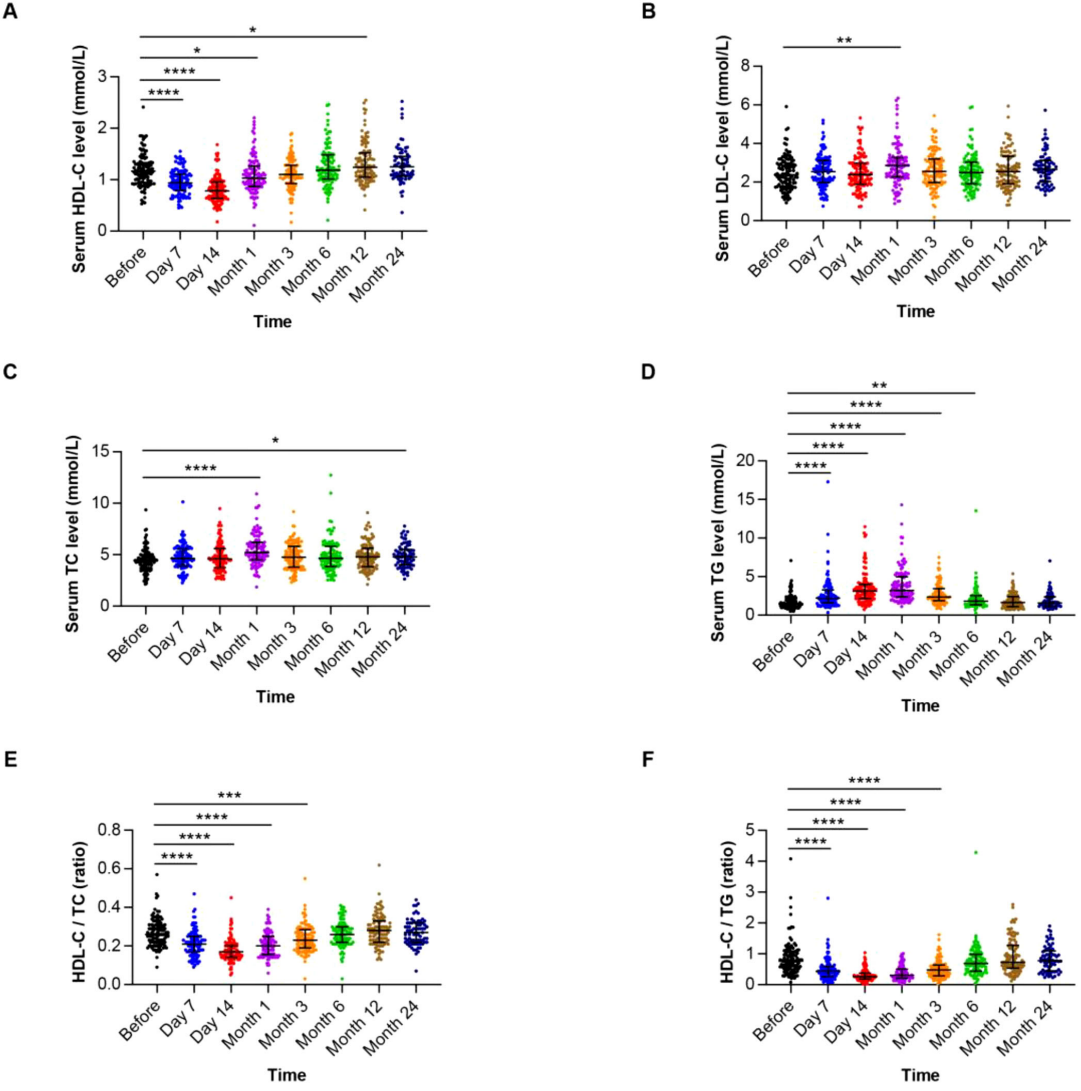
The trajectory of lipid levels during HSCT. **(A)** Showing the levels of high-density lipoprotein-cholesterol (HDL-C) over time. **(B)** Showing the levels of low-density lipoprotein-cholesterol (LDL-C) over time. **(C)** Showing the levels of total cholesterol (TC) over time. **(D)** Showing the levels of triglyceride (TG) over time. **(E)** Showing the HDL-C/TC ratios over time. **(F)** Showing the HDL-C/TG ratios over time. *P<0.05, **P<0.01, ***P<0.001, ****P<0.0001.

To accurately characterize the temporal evolution of serum lipid profiles relative to transplantation, we included 106 patients with complete lipid measurements at predefined time points: pre-transplant (baseline), day 7, day 14, and months 1, 3, 6, 12, and 24 post-HSCT. Analysis revealed significant dynamic changes in lipid levels over time. While a subset of patients exhibited dyslipidemia pre-transplant, a pronounced alteration in lipid profiles became evident post-HSCT. Specifically, many survivors developed significantly lower serum HDL-C and/or higher TG, TC, and LDL-C levels after transplantation compared to baseline (**Figure 1**, **Supplementary Table 1**). The most dramatic shift occurred within the first month post-HSCT, characterized by a steep decline of HDL-C. Notably, HDL-C reached its nadir by day 14, followed by a slow recovery phase. Although a gradual recovery trend in lipid levels emerged after month 1, persistent alterations compared to baseline remained common during long-term follow-up (up to 24 months), particularly for HDL-C. Consequently, the predominant dyslipidemia observed within 3 month post-HSCT featured significantly decreased HDL-C levels and reduced HDL-C/TC and HDL-C/TG ratios. The overall temporal trends and individual patient variations are detailed in **Figure 1** and **Supplementary Table 1**.

### Impact of lipid levels on transplantation outcomes

**Figure 2:**
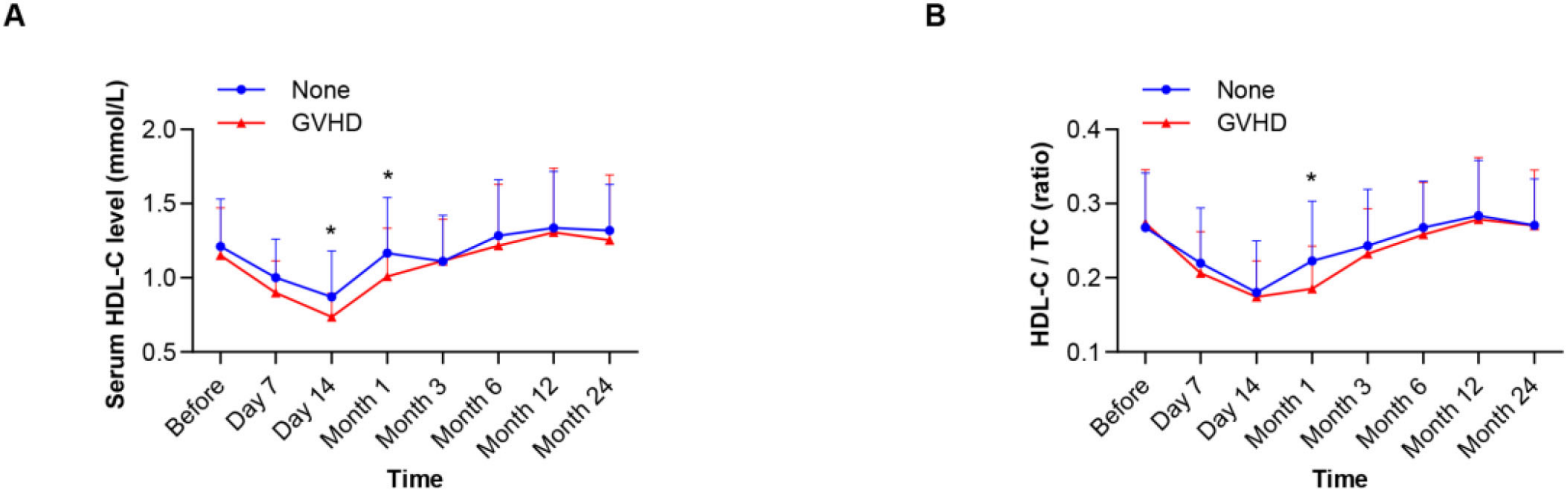
The HDL-C levels and HDL-C/TC ratios in the non-GVHD group and GVHD group during HSCT. **(A)** Showing the levels of HDL-C in the two groups over time. **(B)** Showing the HDL-C/TC ratios in the two groups over time. *P<0.05.

We examined the correlation between lipid levels and the clinical outcomes of HSCT. The median follow‐up of survivors was 34.07 months (range: 0.70–77.47 months). A total of 38 patients (35.85%) had acute GVHD. The incidence of grade I-II aGVHD and grade III-IV aGVHD was 23.58% and 12.26% (**Table 2**), respectively. The incidence of cGVHD was 10.38%. HDL-C and the HDL-C/TC ratio decreased significantly in the GVHD group, especially at 14 days and/or 1 month after transplantation (**Figure 2**). The detailed characteristics of lipid levels are shown in **Supplementary Table 1**.

**Table 2:** Lipid levels of HSCT patients with aGVHD and/or cGVHD.

Lipid markers	None (n=61)	I–II aGVHD (n=25)	III–IV aGVHD (n=13)	cGVHD (n=11)	*P1*	*P2*	*P3*
Before							
HDL-C (mmol/l)	1.18 (0.57-2.41)	1.13 (0.53-1.78)	1.21 (0.60-1.85)	1.13 (0.55-1.85)	0.477	0.782	0.597
HDL-C/TC	0.26 (0.14-0.57)	0.26 (0.19-0.46)	0.33 (0.19-0.47)	0.26 (0.09-0.34)	0.811	0.069	0.874
Day 7							
HDL-C (mmol/L)	0.99 (0.46-1.55)	0.93 (0.48-1.35)	0.85 (0.64-1.10)	0.81 (0.45-1.17)	0.324	0.086	0.142
HDL-C/TC	0.22 (0.09-0.47)	0.20 (0.10-0.31)	0.20 (0.13-0.38)	0.17 (0.12-0.27)	0.209	0.510	0.110
Day 14							
HDL-C (mmol/L)	0.85 (0.18-1.68)	0.78 (0.45-1.11)	0.70 (0.46-1.01)	0.68 (0.46-1.02)	0.174	**0.045**	0.092
HDL-C/TC	0.17 (0.05-0.45)	0.17 (0.06-0.27)	0.18 (0.08-0.29)	0.17 (0.08-0.24)	0.796	0.841	0.948
Month 1							
HDL-C (mmol/L)	1.10 (0.51-2.20)	0.95 (0.46-2.07)	1.03 (0.11-1.63)	1.08 (0.73-1.59)	0.052	0.333	0.297
HDL-C/TC	0.21 (0.11-0.48)	0.16 (0.10-0.29)	0.20 (0.06-0.32)	0.18 (0.14-0.33)	**0.034**	0.408	0.250
Month 3							
HDL-C (mmol/L)	1.08 (0.17-1.90)	1.13 (0.55-1.88)	1.11 (0.71-1.54)	1.09 (0.80-1.26)	0.772	0.940	0.533
HDL-C/TC	0.24 (0.03-0.55)	0.20 (0.16-0.39)	0.24 (0.16-0.29)	0.21 (0.15-0.31)	0.449	0.513	0.338
Month 6							
HDL-C (mmol/L)	1.21 (0.63-2.46)	1.19 (0.82-1.93)	1.17 (0.21-2.44)	1.11 (0.81-1.50)	0.974	0.630	0.320
HDL-C/TC	0.26 (0.13-0.40)	0.24 (0.12-0.36)	0.28 (0.03-0.41)	0.24 (0.14-0.34)	0.277	0.472	0.670
Month 12							
HDL-C (mmol/L)	1.27 (0.41-2.49)	1.13 (0.69-2.55)	1.55 (0.81-2.36)	1.12 (0.83-1.55)	0.163	0.388	0.119
HDL-C/TC	0.29 (0.13-0.43)	0.27 (0.14-0.39)	0.28 (0.17-0.62)	0.26 (0.17-0.42)	0.803	0.999	0.328
Month 24							
HDL-C (mmol/L)	1.25 (0.77-2.28)	1.09 (0.36-2.52)	1.43 (0.68-2.38)	1.15 (0.91-1.45)	0.307	0.324	0.498
HDL-C/TC	0.27 (0.17-0.41)	0.26 (0.07-0.44)	0.31 (0.21-0.42)	0.24 (0.21-0.34)	0.902	0.260	0.880

HDL-C, high-density lipoprotein-cholesterol; TC, total cholesterol; GVHD, graft-versus-host disease; aGVHD, acute graft-versus-host disease; cGVHD, chronic graft-versus-host disease. *P1*: grade I-II aGVHD vs. non-GVHD; *P2*: grade III-IV aGVHD vs. non-GVHD; *P3*: cGVHD vs. non-GVHD.

Values in bold indicate statistical significance at P < 0.05.

**Figure 3:**
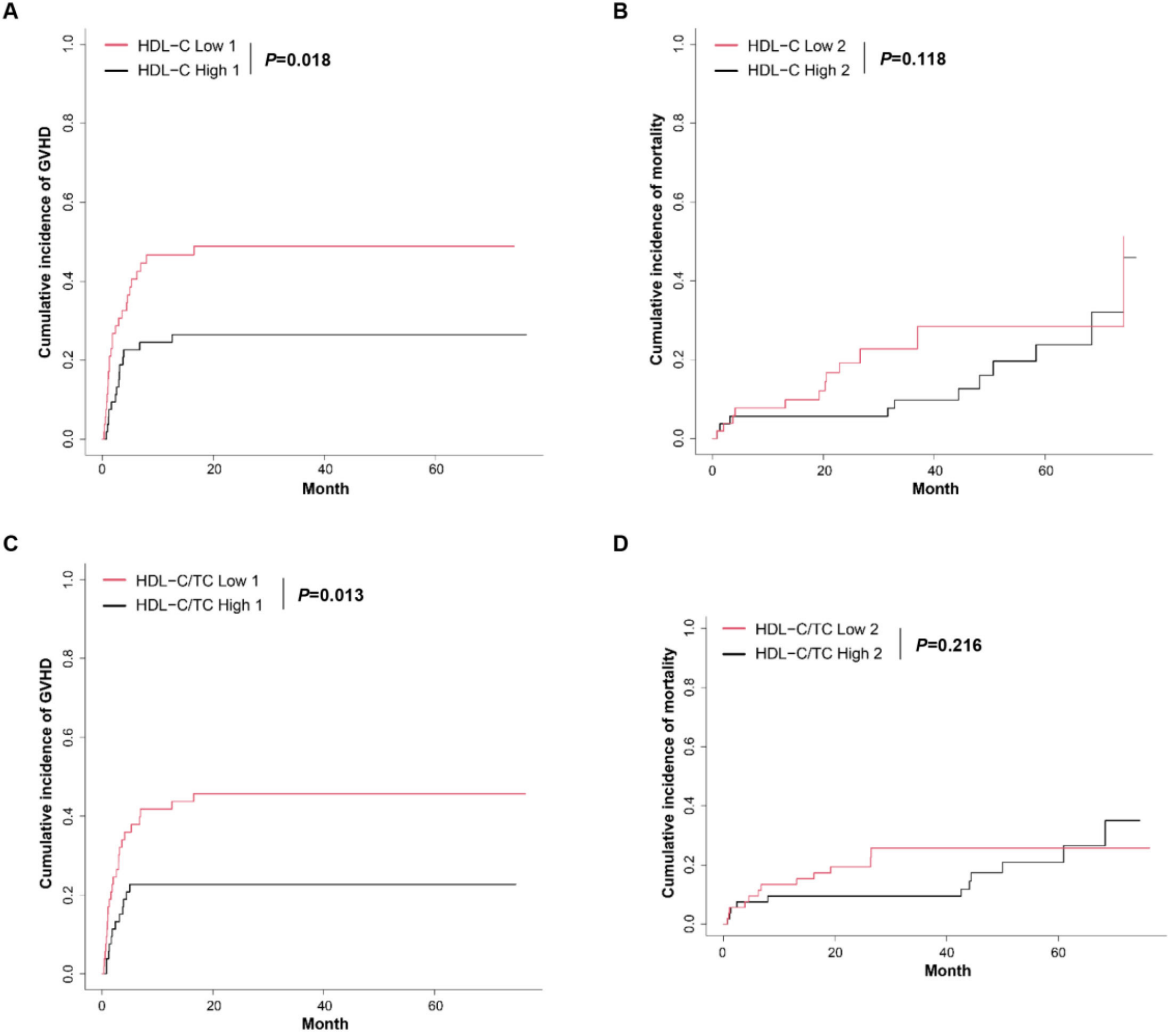
The cumulative incidence of GVHD and mortality during HSCT. **(A)** Competing risks model showing the cumulative incidence of GVHD in the low HDL-C group and high HDL-C group. **(B)** Competing risks model showing the cumulative incidence of mortality in the low HDL-C group and high HDL-C group. **(C)** Competing risks model showing the cumulative incidence of GVHD in the low HDL-C/TC ratio group and high HDL-C/TC ratio group. **(D)** Competing risks model showing the cumulative incidence of mortality in the low HDL-C/TC ratio group and high HDL-C/TC ratio group. HDL-C low: HDL-C levels ≤ 0.78 mmol/L; HDL-C high: HDL-C levels > 0.78 mmol/L; HDL-C/TC low: HDL-C/TC ratio ≤ 0.17, HDL-C/TC high: HDL-C/TC ratio > 0.17. The HDL-C levels and HDL-C/TC ratios were from data of day 14 after transplantation. “1” represents the defined outcome event, which is GVHD; whereas “2” represents competing risks, namely mortality, which includes GVHD-related mortality and non-GVHD-related mortality (e.g. leukemia relapse or graft failure).

Analysis of GVHD outcomes across severity grades (acute grades I-IV and chronic GVHD) revealed that patients with grade III-IV aGVHD exhibited significantly lower serum HDL-C levels at day 14 post-transplantation compared to non-GVHD controls (*P*=0.045). Conversely, those with grade I-II aGVHD demonstrated a markedly reduced HDL-C/TC ratio at 1 month post-HSCT relative to the non-GVHD group (*P*=0.034). No significant differences in lipid profiles were observed between patients with cGVHD and non-GVHD counterparts at any measured timepoint (**Table 2**). Cumulative incidence analysis of GVHD outcomes revealed significantly higher cumulative incidence in patients with low HDL-C levels (≤0.78 mmol/L; *P*=0.018) and a low HDL-C/TC ratio (≤0.17; *P*=0.013), compared to those with high levels (HDL-C levels measured at day 14 post-HSCT). However, no association was observed between HDL-C levels or the HDL-C/TC ratio and overall mortality (including both GVHD-related and non-GVHD-related mortality) (**Figure 3**).

**Figure 4:**
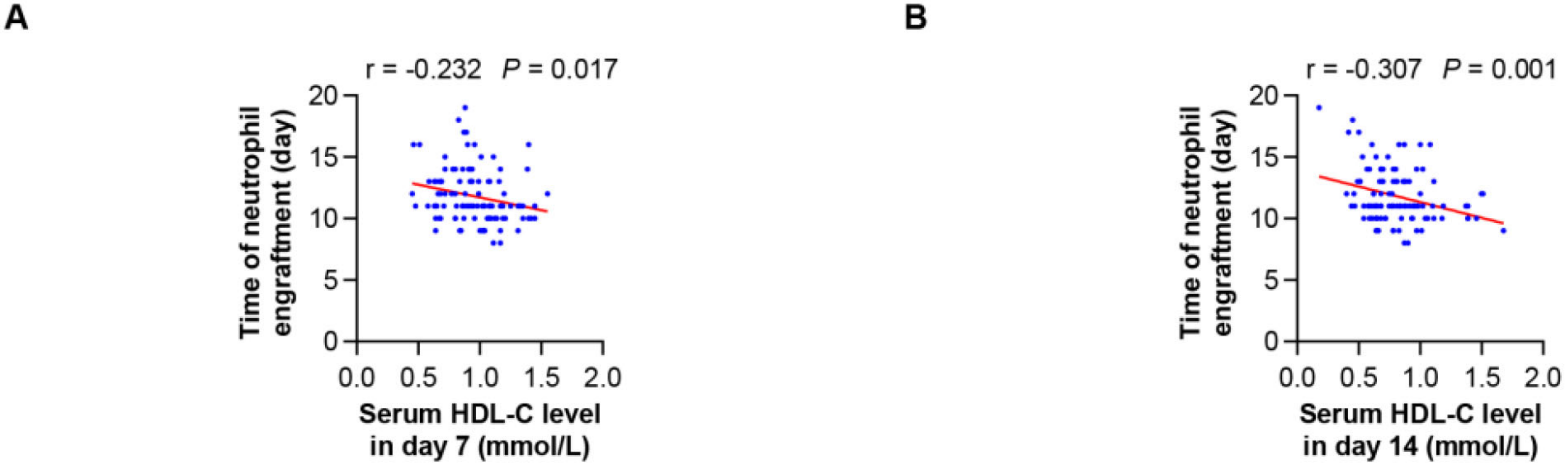
Regression plot of the serum HDL-C levels and the time to neutrophil engraftment for HSCT. **(A)** Showing the correlation between the serum HDL-C levels of day 7 and the time to neutrophil engraftment. **(B)** Showing the correlation between the serum HDL-C levels of day 14 and the time to neutrophil engraftment.

Furthermore, early post-HSCT HDL-C levels (measured at day 7 and day 14 post HSCT) exhibited a significant inverse correlation with time to neutrophil engraftment, indicating that lower HDL-C correlated with delayed myeloid recovery (**Figure 4**).

**Figure 5:**
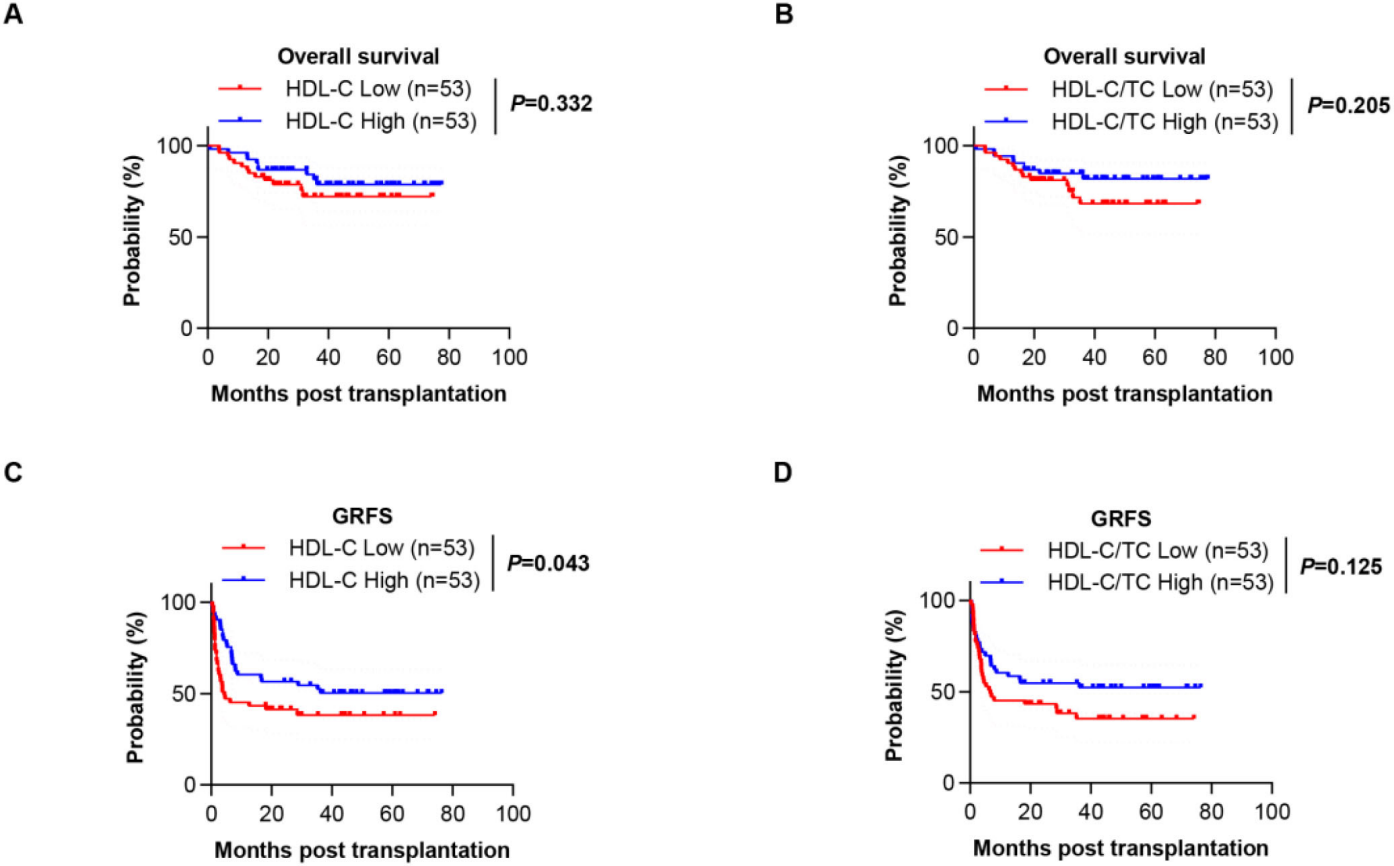
The overall survival (OS) and GVHD-free/relapse-free survival (GRFS) of HSCT patients. **(A)** Kaplan-Meier analysis showing the OS for patients in the low HDL-C group and high HDL-C group. **(B)** Kaplan-Meier analysis showing the OS for patients in the low HDL-C/TC ratio group and high HDL-C/TC ratio group. **(C)** Kaplan-Meier analysis showing the GRFS for patients in the low HDL-C group and high HDL-C group. **(D)** Kaplan-Meier analysis showing the GRFS for patients in the low HDL-C/TC ratio group and high HDL-C/TC ratio group.

Among 106 HSCT recipients, 23 deaths (21.70%) occurred with GVHD complications being the predominant cause (39.13%), followed by relapse (34.78%) and infection (17.39%). The 1-year and 2-year OS rates were 92.5% and 82.9%, respectively, while the 1-year and 2-year GRFS rates were 52.8% and 49.0%. No significant differences in lipid parameters were observed between survivors and non-survivors (*P*>0.05), and neither HDL-C levels nor HDL-C/TC ratio showed association with OS (**Figure 5**). Notably, lower HDL-C levels was significantly associated with worse GRFS outcomes (**Figure 5**). This dichotomy suggests HDL-C’s protective effect manifests primarily through GVHD mitigation rather than overall mortality reduction in this cohort.

### Impact of HDL-C trajectories on GVHD Risk after HSCT

**Figure 6:**
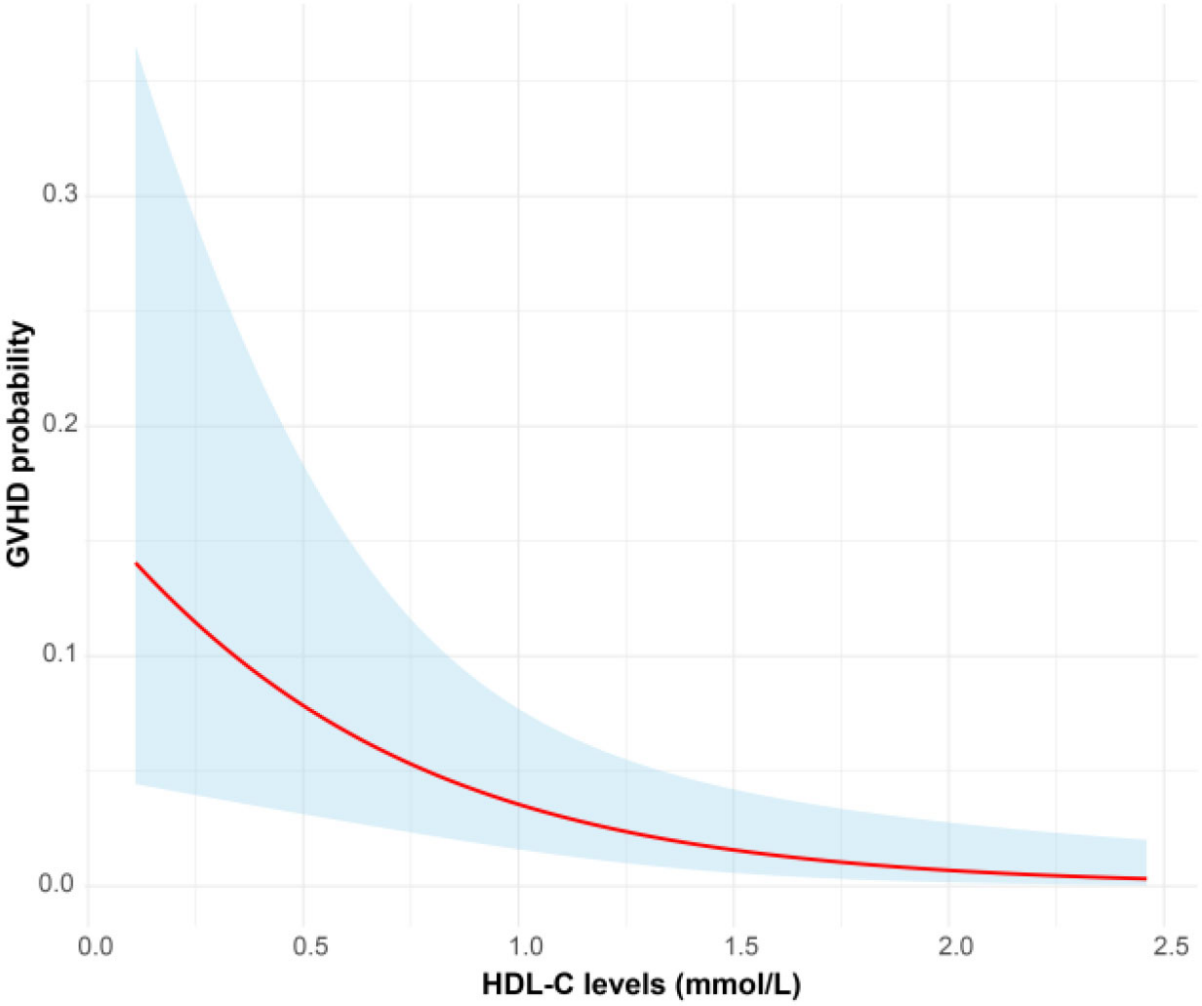
Longitudinal data analysis showing the relationship of HDL-C level changes with the development probability of GVHD.

To better elucidate the relationship between HDL-C levels and GVHD, we employed generalized linear mixed models (GLMM) to investigate the dynamic effects of HDL-C levels and post-transplantation time on the probability of GVHD occurrence. The GLMM analysis demonstrated a significant interaction between HDL-C levels and GVHD, indicating that the protective effect of HDL-C varies over the post-HSCT course (**Figure 6**). The 3D surface plot (**Supplementary Figure 1**) quantified HDL-time effects on GVHD probability. HDL-C <1.0 mmol/L within 6 months post-HSCT caused steep risk elevation. At HDL-C=0.6 mmol/L (Month 1), probability peaked at 12.0%. Levels ≥1.8 mmol/L maintained risk < 4.0%.

**Figure 7:**
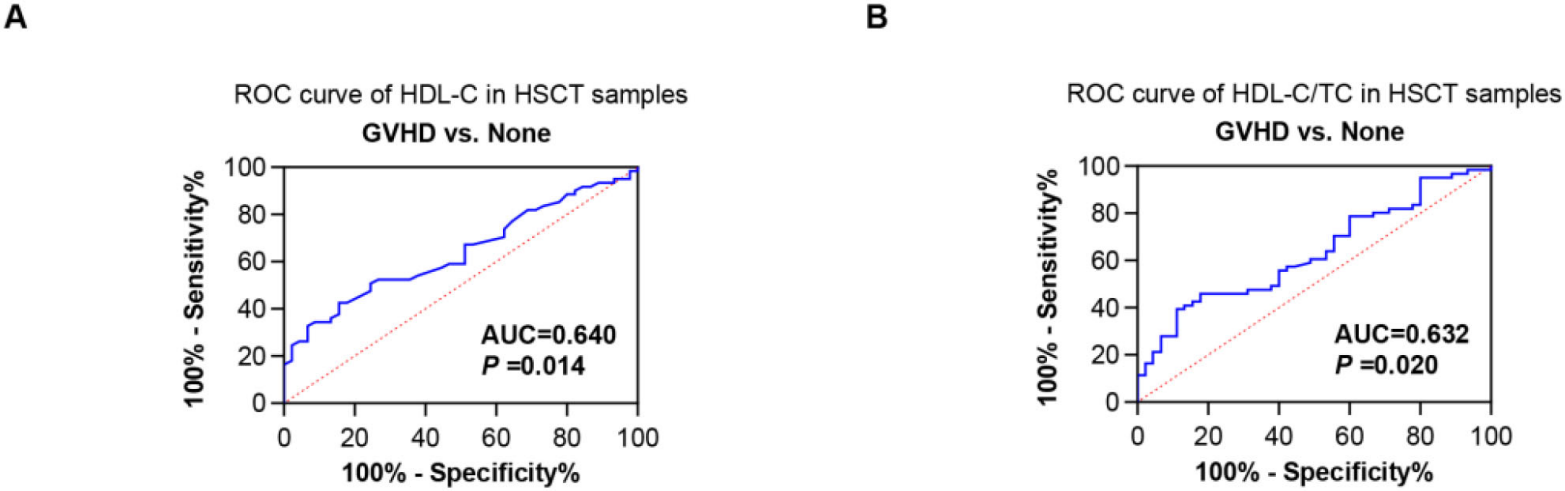
ROC curves of the utility of the HDL-C levels and HDL-C/TC ratios for predicting GVHD. **(A)** ROC curves showing the diagnostic efficiency of HDL-C levels for GVHD. **(B)** ROC curves showing the diagnostic efficiency of HDL-C/TC ratios for GVHD.

Next, the utility of the HDL-C level and HDL-C/TC ratio (day 14 post-transplantation) for predicting GVHD were estimated. The AUC for the HDL-C level was 0.640 (*P* =0.014), and the AUC for the HDL-C/TC ratio was 0.632 (*P* =0.02) (**Figure 7**). Univariate analysis found that HDL-C ≤ 0.84 mmol/L was risk factors for GVHD. Further multivariate analysis showed that HDL-C ≤ 0.84 mmol/L was independent risk factors for GVHD [OR: 3.15, 95% CI, 1.20-8.26, *P*=0.020] (**Table 3**). Therefore, lower HDL-C levels could be simple biochemical markers of GVHD in HSCT. These findings suggest that HDL monitoring may be particularly valuable for GVHD risk stratification during the early post-transplantation period when its protective effects are most pronounced. The time-dependent nature of this relationship highlights the importance of considering both HDL levels and temporal factors in clinical assessment.

**Table 3:** Risk associations between HDL-C levels and GVHD.

Lipid	Unadjusted odds ratio (95% CI)	*P* value	Adjusted odds ratio* (95% CIs)	*P* value
HDL-C				
>0.84 mmol/L	1	**0.006**	1	**0.020**
≤ 0.84 mmol/L	3.19 (1.35-7.24)		3.15 (1.20-8.26)	
HDL-C/TC				
>0.19	1	0.199	1	0.656
≤ 0.19	1.71 (0.73-4.02)		1.25 (0.47-3.30)	

The data are presented as odds ratios (ORs) and 95% confidence intervals.

*Adjusted for sex, age and diagnosis (including acute lymphoid leukemia, acute myeloid leukemia, aplastic anemia, myelodysplastic syndrome, and other diseases).

Values in bold indicate statistical significance at P < 0.05.

## Discussion

This study aimed to directly describe serum lipid level changes during the course of HSCT, and the results provide critical insights into the role of lipid levels in GVHD. We found a characteristic pattern of changes in lipid profiles during HSCT. Almost 60%~80% of the patients had at least one kind of dyslipidemia during the course of HSCT, with most patients showing decreased HDL levels within 1 month after HSCT. Interestingly, serum HDL-C levels were associated with GVHD in HSCT patients and could predict the presence of GVHD. In addition, a lower HDL-C was independently associated with GVHD. Our findings indicate that decreased HDL-C levels may play a role in the development of graft-versus-host immune effects.

Similar to the findings of our study, HSCT recipients have been reported to exhibit abnormal lipid levels in other papers. Several studies have reported the incidence of posttransplantation dyslipidemia to range from 39% to 80% (12, 13). The incidence of hypercholesterolemia and hypertriglyceridemia in the first 2 years after transplantation was 73.4% and 72.5%, respectively (14). Dyslipidemia is being increasingly recognized as a complication of allogeneic HSCT. Nonetheless, the association between lipid levels and the clinical outcomes of HSCT have rarely been studied. Here, we conducted a clinical study and found that reduced HDL-C levels were associated with GVHD. Additionally, Chagué et al. demonstrated that repeated administration of HDL isolated from human plasma significantly decreased the mortality and severity of aGVHD in recipient model mice (6), which indicated the potential therapeutic value of HDL in the prevention of aGVHD.

While direct mechanistic evidence linking HDL to GVHD pathogenesis remains limited, our findings demonstrating a significant protective association align with established immunomodulatory properties of HDL-C (15). Critically, simple interventions capable of elevating HDL-C—including statins (16), low-carbohydrate diets (17), and supervised exercise (18)—represent readily implementable strategies. Whether these HDL-centric interventions directly reduce GVHD incidence remains a pivotal research question.

Our data shed light on the relationship between the lipid profile and GVHD during HSCT. However, there are several limitations that need to be addressed. First, the study is limited by the size of the cohort, and this was a retrospective cross-sectional study, which limits the possibility of exploring a causal relationship between the lipid profile and transplantation outcomes. Second, systematic lipid measurements during therapy were not taken for all patients in the study population, which limits the comprehensive evaluation of lipid profiles. Third, the effects of drugs are complex and were not considered in our study, but immunosuppressive regimens have been documented to play a crucial role in lipid metabolism. Future research should include consideration of these drug effects to further confirm that the lipid profile is independently associated with transplantation outcomes.

In conclusion, our study showed that lower HDL-C levels were associated with a higher incidence of GVHD. Clinical manifestations of altered HDL-C levels need to be recognized independently. Further prospective evaluation of lipid abnormalities is thus warranted.

## Data Availability

The original contributions presented in the study are included in the article/**Supplementary Material**. Further inquiries can be directed to the corresponding authors.
