# Supplementary figures and images for: Lipid biomarkers of GVHD in allogeneic stem hematopoietic cell transplantation patients
Source: Front Immunol. 2025 Sep 2;16:1624168. doi: 10.3389/fimmu.2025.1624168 (PMC12436127; doi:10.3389/fimmu.2025.1624168)

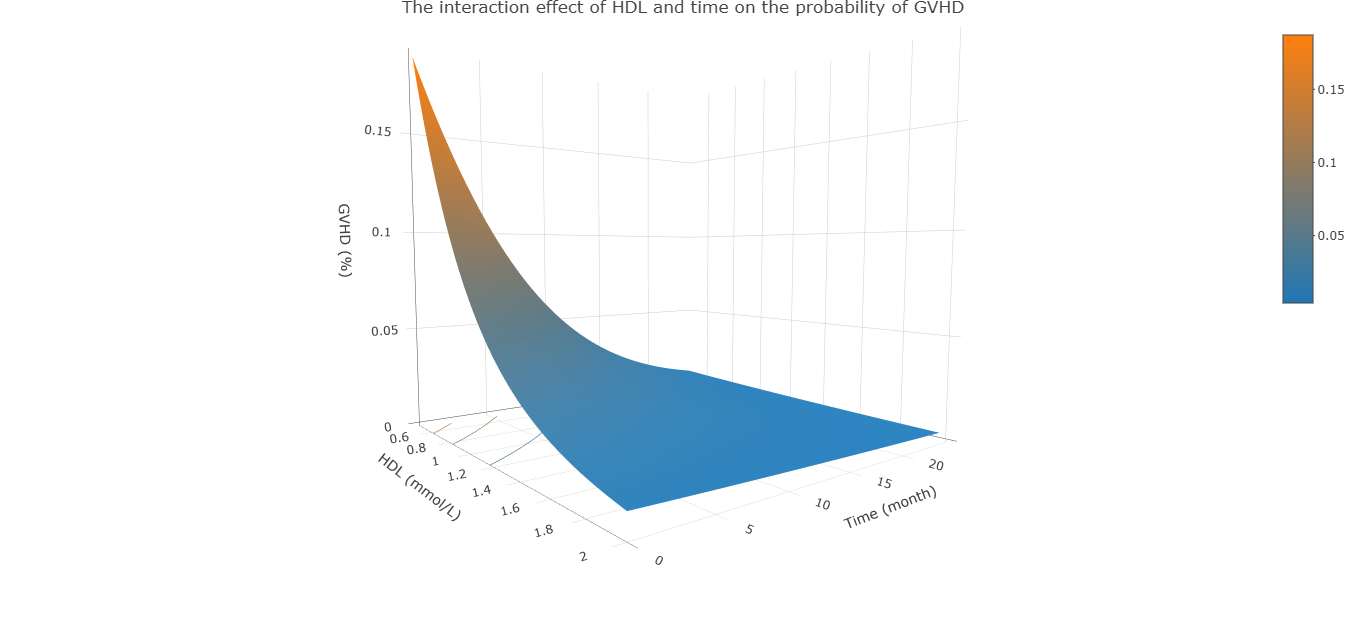

Supplement: Supplementary file 1 [file Image1.png]
