# Supplementary material for: Lipid biomarkers of GVHD in allogeneic stem hematopoietic cell transplantation patients
Source: Front Immunol. 2025 Sep 2;16:1624168. doi: 10.3389/fimmu.2025.1624168 (PMC12436127; doi:10.3389/fimmu.2025.1624168)
Supplement: Supplementary file 2 [file Table1.docx]

**Supplementary Table 1. Lipid levels of HSCT patients with and without GVHD**

| **Lipid markers** | **Total (n=106)** | **None (n=61)** | **GVHD (n=45)** | ***P*** |
| --- | --- | --- | --- | --- |
| **Before** |  |  |  |  |
| HDL-C (mmol/L) | 1.17 (0.53-2.41) | 1.18 (0.57-2.41) | 1.16 (0.53-1.85) | 0.614 |
| LDL-C (mmol/L) | 2.38 (0.93-5.92) | 2.38 (0.93-5.92) | 2.29 (1.16-4.01) | 0.616 |
| TC (mmol/L) | 4.45 (2.15-9.37) | 4.59 (2.43-9.37) | 4.31 (2.15-6.35) | 0.294 |
| TG (mmol/L) | 1.49 (0.50-7.07) | 1.53 (0.50-4.01) | 1.45 (0.80-7.07) | 0.583 |
| HDL-C/TC | 0.26 (0.09-0.57) | 0.26 (0.14-0.57) | 0.26 (0.09-0.47) | 0.476 |
| **Day 7** |  |  |  |  |
| HDL-C (mmol/L) | 0.94 (0.45-1.55) | 0.99 (0.46-1.55) | 0.90 (0.45-1.35) | 0.091 |
| LDL-C (mmol/L) | 2.55 (0.75-5.20) | 2.52 (1.08-5.20) | 2.57 (0.75-4.37) | 0.662 |
| TC (mmol/L) | 4.63 (2.25-10.10) | 4.61 (2.25-10.10) | 4.64 (2.56-6.52) | 0.555 |
| TG (mmol/L) | 2.21 (0.35-17.30) | 2.15 (0.35-17.30) | 2.25 (1.01-10.50) | 0.716 |
| HDL-C/TC | 0.21 (0.09-0.47) | 0.22 (0.09-0.47) | 0.20 (0.10-0.38) | 0.173 |
| **Day 14** |  |  |  |  |
| HDL-C (mmol/L) | 0.78 (0.18-1.68) | 0.85 (0.18-1.68) | 0.75 (0.45-1.11) | **0.028** |
| LDL-C (mmol/L) | 2.38 (0.73-5.33) | 2.48 (1.10-5.33) | 2.31 (0.73-4.01) | 0.110 |
| TC (mmol/L) | 4.58 (2.64-9.49) | 4.81 (2.64-9.49) | 4.50 (2.65-7.83) | 0.075 |
| TG (mmol/L) | 3.12 (0.75-11.50) | 3.16 (1.03-10.70) | 3.03 (0.75-11.50) | 0.963 |
| HDL-C/TC | 0.17 (0.05-0.45) | 0.17 (0.05-0.45) | 0.17 (0.06-0.29) | 0.850 |
| **Month 1** |  |  |  |  |
| HDL-C (mmol/L) | 1.03 (0.11-2.20) | 1.10 (0.51-2.20) | 0.98 (0.11-2.07) | **0.027** |
| LDL-C (mmol/L) | 2.86 (0.89-6.35) | 2.87 (0.89-5.80) | 2.68 (1.02-6.35) | 0.637 |
| TC (mmol/L) | 5.22 (1.87-10.90) | 5.23 (2.94-10.90) | 5.22 (1.87-9.76) | 0.766 |
| TG (mmol/L) | 3.23 (1.13-14.30) | 3.11 (1.13-10.10) | 3.40 (1.60-14.30) | 0.634 |
| HDL-C/TC | 0.20 (0.06-0.48) | 0.21 (0.11-0.48) | 0.18 (0.06-0.33) | **0.029** |
| **Month 3** |  |  |  |  |
| HDL-C (mmol/L) | 1.10 (0.17-1.90) | 1.08 (0.17-1.90) | 1.11 (0.55-1.88) | 0.988 |
| LDL-C (mmol/L) | 2.55 (0.18-5.44) | 2.55 (0.18-4.66) | 2.55 (0.72-5.44) | 0.800 |
| TC (mmol/L) | 4.77 (2.31-9.20) | 4.66 (2.31-9.20) | 4.92 (2.46-7.77) | 0.357 |
| TG (mmol/L) | 2.36 (0.81-7.52) | 2.36 (0.85-7.52) | 2.36 (0.81-6.10) | 0.768 |
| HDL-C/TC | 0.23 (0.03-0.55) | 0.24 (0.03-0.55) | 0.22 (0.15-0.39) | 0.335 |
| **Month 6** |  |  |  |  |
| HDL-C (mmol/L) | 1.19 (0.21-2.46) | 1.21 (0.63-2.46) | 1.18 (0.21-2.44) | 0.469 |
| LDL-C (mmol/L) | 2.48 (1.06-5.90) | 2.50 (1.15-5.90) | 2.44 (1.06-5.85) | 0.930 |
| TC (mmol/L) | 4.66 (2.52-12.70) | 4.65 (2.56-11.00) | 4.71 (2.52-12.70) | 0.834 |
| TG (mmol/L) | 1.80 (0.24-13.60) | 1.78 (0.94-4.86) | 1.80 (0.24-13.60) | 0.710 |
| HDL-C/TC | 0.26 (0.03-0.41) | 0.26 (0.13-0.40) | 0.27 (0.03-0.41) | 0.608 |
| **Month 12** |  |  |  |  |
| HDL-C (mmol/L) | 1.24 (0.41-2.55) | 1.27 (0.41-2.49) | 1.17 (0.69-2.55) | 0.424 |
| LDL-C (mmol/L) | 2.54 (0.80-5.94) | 2.46 (1.17-5.94) | 2.62 (0.80-5.37) | 0.626 |
| TC (mmol/L) | 4.80 (2.12-9.10) | 4.70 (3.06-8.11) | 4.82 (2.12-9.10) | 0.799 |
| TG (mmol/L) | 1.64 (0.71-5.38) | 1.55 (0.71-4.46) | 1.68 (0.71-5.38) | 0.243 |
| HDL-C/TC | 0.28 (0.13-0.62) | 0.29 (0.13-0.43) | 0.27 (0.14-0.62) | 0.651 |
| **Month 24** |  |  |  |  |
| HDL-C (mmol/L) | 1.25 (0.36-2.52) | 1.25 (0.77-2.28) | 1.17 (0.36-2.52) | 0.523 |
| LDL-C (mmol/L) | 2.66 (1.33-5.73) | 2.78 (1.55-4.70) | 2.58 (1.33-5.73) | 0.225 |
| TC (mmol/L) | 4.80 (2.64-7.79) | 4.82 (3.08-7.23) | 4.76 (2.64-7.79) | 0.397 |
| TG (mmol/L) | 1.58 (0.73-7.05) | 1.58 (0.73-4.20) | 1.56 (0.81-7.05) | 0.744 |
| HDL-C/TC | 0.27 (0.07-0.44) | 0.27 (0.17-0.41) | 0.26 (0.07-0.44) | 0.735 |

HDL-C, high-density lipoprotein-cholesterol; LDL-C, low-density lipoprotein-cholesterol; TC, total cholesterol; TG, triglyceride.
